# Area deprivation index, a marker of socioeconomic disadvantage, may predict severity of COVID-19 in patients and which families may experience worse symptoms of PTSD, anxiety, and depression post-ICU

**DOI:** 10.1186/s44158-024-00198-8

**Published:** 2024-09-19

**Authors:** Megan Mayer, Meghan Althoff, Nicholas Csikesz, Stephanie Yu, Hope Cruse, Renee Stapleton, Timothy Amass

**Affiliations:** 1 University of Washington, 3518 Fremont Ave N #495, Seattle, WA 98103 USA; 2 University of Colorado, Aurora, CO USA; 3 South Shore Health, Weymouth, MA USA; 4 Mayo Clinic, Phoenix, AZ USA; 5 University of Vermont, Burlington, VT USA

**Keywords:** COVID-19, ICU experience, Area deprivation index, Social determinants of health

## Abstract

**Background:**

COVID-19 disproportionately impacted marginalized populations early in the pandemic. Families of patients admitted to the intensive care unit (ICU) experienced significant psychological effects. Little is known about whether individual and patient psychological outcomes after a loved ones stay in the ICU differs by socioeconomic status, as measured by the area deprivation index (ADI).

**Methods:**

Family members of patients with COVID-19 respiratory failure admitted to the ICU at twelve hospitals in five US states were enrolled in a larger study looking at rates of symptoms of post-traumatic stress disorder (PTSD), anxiety, and depression in the months following their loved one's ICU stay. This secondary data analysis includes eight of the twelve hospitals in four of the five states. Each participant was assigned a number indicating a level of neighborhood disadvantage based on the patient’s zip code. Patient and family level characteristics as well as symptoms of anxiety, depression, and PTSD were assessed among each neighborhood.

**Results:**

Patients from the most disadvantaged neighborhoods had the highest proportion of patients that needed to be intubated (*p* = 0.005). All the patients in the most disadvantaged neighborhoods were a race other than white (*p* = 0.17). At 12 months post-hospitalization, there was a statistically significant difference in the proportion of family members who experienced symptoms of PTSD, anxiety, and depression between the ADI groups.

**Conclusions:**

ADI may be a predictor of COVID-19 disease severity for patients on presentation to the ICU. Patients and family members experience psychological effects after a loved one’s admission to the ICU, and these outcomes vary among individuals of different socioeconomic status’, as measured by the ADI. A larger study of family members’ incidence of anxiety, depression, and post-traumatic stress disorder is needed to understand the extent to which these symptoms are impacted by neighborhood level factors as measured by the ADI.

## Background

Family members of patients in the intensive care unit (ICU) can experience a variety of stressful and traumatic events that can cause anxiety, depression, stress, and post-intensive care syndrome-family (PICS-f) [1]. This was heightened in the early COVID-19 pandemic as families of critically ill patients were unable to visit their loved ones in the hospital leading to increased symptoms of post-traumatic stress disorder (PTSD) among family members of ICU patients with COVID-19 [2].

Efforts to understand how social determinants of health impact individual health in the United States are ongoing [3]. People of lower socioeconomic status (SES) often have less access to care [4], shorter life-expectancies [5] and more comorbidities, such as type 2 diabetes [6]. The COVID-19 pandemic also disproportionately impacted disadvantaged communities and highlighted health disparities among racial and ethnic minority groups [7]. Patients from the most disadvantaged neighborhoods experienced higher mortality independent of race [8], highlighting the importance of neighborhood factors that influence health. It is unknown whether the mental health impacts of having a critically ill family member during the COVID-19 pandemic were more pronounced in families coming from disadvantaged neighborhoods.

The Area Deprivation Index (ADI) is a census-block-level composite measurement that assigns a socioeconomic disadvantage score to certain neighborhoods that are defined by census data [9, 10] and can be used as a surrogate for social determinants of health. The factors that go into the ADI include but are not limited to income, education, employment, and housing quality [9, 10]. The ADI of a particular area can be used to compare health outcomes among patients beyond individual level characteristics.

We sought to understand if ADI could correlate with disease severity at presentation for patients with COVID-19 and whether the development of symptoms of anxiety, depression, and post-traumatic stress disorder (PTSD) in family members of patients admitted to the ICU with COVID-19 differed by SES, as measured by the Area Deprivation Index (ADI) of the patient.

## Methods

This is a secondary data analysis of a prospective cohort study aimed at understanding symptoms of PTSD, anxiety, and depression in families of COVID-19 ICU patients [2]. Family members of patients with COVID-19 respiratory failure admitted to the ICU were enrolled from twelve hospitals in five US states [2]. Calls were made at three, six, and twelve months after their family member’s admission to the ICU to assess for participant symptoms of anxiety, depression, and post-traumatic stress disorder [11]. For this secondary analysis, eight hospitals agreed to participate. Participants from the eight hospitals were assigned an ADI based on the patient’s nine-digit zip code. Any participants without a nine-digit zip code or no accompanying ADI were excluded (*n* = 6). Participants’ ADIs were further subclassified into quintiles (1 = most advantaged, 5 = least advantaged). The outcomes of interest were symptoms of PTSD measured by the impact of events scale 6 (IES-6) and symptoms of anxiety measured by the hospital anxiety and depression scale (HADS). IRB approval was obtained for the parent study, registration number NCT04476914, by Colorado Multiple Institutions Review Board, University of Colorado Anschutz Medical Campus, protocol # 20–1021, titled "Stress Related Disorders in Family Members of COVID19 Patients Admitted to the Intensive Care Unit: A MultiSite, Mixed- Methods Study". All ethical standards regarding human participation in this study were followed and all procedures were in accordance with the Helsinki Declaration. IRB approval date for this secondary data analysis was ​10/4/21, and we requested a full waiver of consent, which was granted. Data was stored securely in RedCap and de-identified for analysis. Bivariate analyses of family and patient demographics as well as symptoms of anxiety, depression and PTSD among family members in the different ADI quintiles were calculated using Fisher’s exact test for dichotomous variables and for non-normally distributed continuous variables (eg. Age), the median values and IQR were used followed by Kruskal–Wallis tests to assess the statistical significance of any difference, reported as the *p*-value. Charlson scores were grouped into categories for clinical interpretation and were compared using Fisher exact tests.

## Results

Among the 164 family members interviewed and included in this analysis, there were 46, 84, 24, 5, and 5 patients in ADIs 1–5, respectively. Baseline patient characteristics did not differ significantly by ADI (Table 1). All the patients in the most disadvantaged neighborhoods were a race other than white (*p* = 0.17). While the highest Charlson scores were among patients from one the most disadvantaged neighborhoods (quintile four predominantly), there were no patients from the most disadvantaged neighborhood (quintile 5) with this same score (*p* = 0.66) despite the fact that 100% of those from quintile 5 had to be intubated (*p* = 0.005). At three months post-hospitalization, 49% of family members met criteria for symptoms of anxiety, 32% for symptoms of depression, and 65% for symptoms of PTSD. At least 40% of family members from each patient ADI group reported symptoms of PTSD at three months, with the highest proportion of individuals experiencing symptoms of PTSD in the fourth, more disadvantaged, quintile (100%, *p* = 0.04) (Table 2a). In addition, at 12 months, 75% of participants in quintile four (more disadvantaged) experienced symptoms of PTSD compared with 15% in quintile 1 (least disadvantaged) (*p* = 0.01). There was a statistically significant difference in symptoms of anxiety among the different quintiles at 12 months only and the largest proportion of individuals experiencing these symptoms were in quintile 4 (*p* = 0.05) (Table 2b). For symptoms of depression, we found that the largest proportion of individuals experiencing symptoms of depression at 12 months were from quintile 3, which correlates with neither advantaged nor disadvantaged (*p* = 0.03) (Table 2c).

**Table 1:** Patient characteristics by ADI

Outcomes of Interest	ADI					*P*-value (2-sided)
N (%)	1 (*n* = 46)	2 (*n* = 84)	3 (*n* = 24)	4 (*n* = 5)	5 (*n* = 5)	
Age (median, IQR)	68 (18)	65 (22)	60 (22)	78 (16)	56 (14)	0.17
Race						
White	21 (46)	29 (34)	7 (29)	3 (60)	0 (0)	0.17
Non-white	25 (54)	55 (66)	17 (71)	2 (40)	5 (100)	
Ethnicity						
Hispanic	15 (35)	35 (44)	7 (35)	2 (40)	1 (33)	0.89
Non-Hispanic	27 (65)	44 (56)	13 (65)	3 (60)	2 (67)	
Charlson score^a^						
Low risk	15 (33)	27 (32)	9 (38)	1 (20)	2 (40)	0.66
Mild	21 (45)	39 (46)	9 (38)	1 (20)	2 (40)	
Moderate	5 (11)	9 (11)	2 (8)	0 (0)	1 (20)	
Severe	5 (11)	9 (11)	4 (16)	3 (60)	0 (0)	
Days in ICU (median, IQR)	10 (18)	14 (22)	12 (22)	2 (16)	15 (14)	0.27
COVID severity (IMV^b^)						
Never intubated	12 (26)	11 (13)	4 (17)	4 (80)	0 (0)	0.005
Intubated	34 (74)	73 (87)	19 (79)	1 (20)	5 (100)	
Missing	0 (0)	0 (0)	1 (4)	(0)	(0)	
SOFA score on admission (median, IQR)	8 (8)	10 (6)	11 (8)	9 (3)	11 (1)	0.17

^a^Charlson score: low risk (score 0), mild (score 1–2), moderate (score 3–4), severe (score > 4)

^b^*IMV* invasive mechanical ventilation

**Table 2:** (a, b, c): PTSD, Anxiety, and Depression among family members by ADI

a. PTSD among family members	ADI					*p*-value (2-sided)
	1 N (%)	2 N (%)	3 N (%)	4 N (%)	5 N (%)	
3 month						
No	14 (30)	37 (45)	5 (21)	0 (0)	3 (60)	0.04
Yes	32 (70)	45 (55)	19 (79)	5 (100)	2 (40)	
6 month						
No	18 (64)	27 (55)	7 (41)	0 (0)	2 (100)	0.11
Yes	10 (36)	22 (45)	10 (59)	3 (100)	0 (0)	
12 month						
No	23 (85)	31 (66)	5 (42)	1 (25)	1 (100)	0.01
Yes	4 (15)	16 (34)	7 (58)	3 (75)	0 (0)	
b. Anxiety	1 N (%)	2 N (%)	3 N (%)	4 N (%)	5 N (%)	*p*-value (2-sided)
3 month						
No	25 (54)	42 (51)	9 (38)	2 (40)	4 (80)	0.44
Yes	21 (46)	40 (49)	15 (62)	3 (60)	1 (20)	
6 month						
No	17 (61)	31 (63)	9 (53)	2 (67)	2 (100)	0.85
Yes	11 (39)	18 (37)	8 (47)	1 (33)	0 (0)	
12 month						
No	18 (67)	32 (68)	6 (50)	0(0)	1 (100)	0.05
Yes	9 (33)	15 (32)	6 (50)	4 (100)	0 (0)	
c. Depression	1 N (%)	2 N (%)	3 N (%)	4 N (%)	5 N (%)	*p*-value (2-sided)
3 month						
No	33 (72)	52 (63)	16 (70)	2 (40)	5 (100)	0.30
Yes	13 (28)	30 (37)	7 (30)	3 (60)	0 (0)	
6 month						
No	23 (82)	36 (74)	11 (65)	2 (67)	2 (100)	0.64
Yes	5 (18)	13 (26)	6 (35)	1 (33)	0 (0)	
12 month						
No	24 (89)	35 (75)	5 (42)	3 (75)	1 (100)	0.03
Yes	3 (11)	12 (26)	7 (58)	1 (25)	0 (0)	

Overall, we found significant family member symptoms of PTSD, anxiety and depression following COVID-19 ICU admissions, with some differences between ADI depending on the follow-up month and symptom being assessed.

## Discussion

This study found that the highest proportion of individuals needing to be intubated was among the most disadvantaged neighborhood quintile (100%) compared with the least disadvantaged neighborhood quintiles (*p* = 0.005), highlighting higher disease severity in that group. This builds on another study that also sought to assess neighborhood factors beyond individual level characteristics for COVID-19 disease severity and found that being from the most disadvantaged neighborhood quintile predicted in-hospital mortality from COVID-19 [12]. Beyond COVID-19, more recent research shows that neighborhood level characteristics also impact individuals’ 30-day mortality after admission to the ICU with severe sepsis, as mortality was higher for patients from the most disadvantaged neighborhoods [13]. This builds on existing literature and understanding that there are social, economic, and living conditions beyond individual level characteristics that impact individuals’ health outcomes, especially as it relates to critical illness. To our knowledge, this is the first study looking at neighborhood disadvantage (ADI) and disease severity of COVID-19 at hospitalization, as well as family experience in the ICU. At the 12 month follow-up, we found that there were statistically significant differences in rates of symptoms of PTSD, anxiety, and depression among the ADI quintile groups. With regard to depression, the highest proportion of symptoms was neither in the most disadvantaged nor in the least disadvantaged quintiles (Table 2c). The reason for this is not fully understood but warrants further investigation and may have been due to incidence of intubations among the patients. These findings may also be due to the small sample size in quintile 5. Additional studies that evaluate ICU experience among various ADIs of patients with all critical illness outside of COVID-19, and their families’ experiences, are needed to begin to understand who experiences higher rates of symptoms of anxiety, depression, and PTSD so we can actively work to combat this issue.

Our study was limited by few participants in high disadvantage neighborhoods and small sample size. In addition, participants were assigned an ADI based on the patients' addresses as a proxy for the participants' as we did not have the participants’ addresses. Multivariate modeling and adjusting for confounders, was not possible due to small sample size. For example, disease severity among patients (such as incidence of intubation among patients) may have impacted the incidence of the outcomes assessed in the participants. This study is purely descriptive, and larger sample sizes aimed at understanding the intersection between ADI, disease severity, and mental health outcomes would help to further elucidate this relationship.

## Conclusion

ADI is a comprehensive tool that can be a surrogate for social determinants of health and was associated with some patient and family factors in this study. It builds on current research that highlights SES as a risk factor for challenges in accessing care and poor patient outcomes. While there were overall high levels of anxiety, depression, and PTSD symptoms among family members of COVID-19 patients, we report significant differences based on patient ADI quintiles in all three stress disorders at the 12 month follow-up. Utilizing a national sample from a larger cohort would help to understand if mental health symptoms of family members of critically ill patients, with COVID-19 or another critical illness, differ by SES.

## Data Availability

Data obtained with PHI was stored in Redcap. Data was de-identified when assigning area deprivation index scores to each participant. Data can be provided upon request.
